# Laser-Induced Surface Modification of Graphene-Modified KM2-600 Para-Aramid Fabrics: Morphological and Topographical Analysis

**DOI:** 10.3390/ma19102078

**Published:** 2026-05-15

**Authors:** Jēkabs Lapa, Ieva Baķe, Imants Adijāns, Silvija Kukle, Uģis Briedis, Ērika Teirumnieka, Lyubomir Lazov

**Affiliations:** 1 Rezekne Academy, Riga Technical University, LV-1658 Riga, Latvia; imants.adijans@rtu.lv (I.A.); erika.teirumnieka@rtu.lv (Ē.T.); lyubomir.lazov@rtu.lv (L.L.); 2 Institute of Architecture and Design, Riga Technical University, LV-1658 Riga, Latvia; ieva.bake@rtu.lv (I.B.); silvija.kukle@rtu.lv (S.K.); ugis.briedis@rtu.lv (U.B.)

**Keywords:** ballistic fabrics, graphene, Kevlar^®^ KM2 fabric (600 dtex), laser processing, para-aramid, surface morphology, surface roughness

## Abstract

Ballistic para-aramid fabrics are widely used in personal protection and defense applications due to their high strength-to-weight ratio, thermal stability, and durability. This study investigates the influence of laser-based surface modification on graphene-modified Kevlar^®^ KM2-600 (600 dtex) fabrics, with a particular focus on surface morphology and topographical characteristics of para-aramid fabrics used in ballistic applications. The deposition of graphene onto para-aramid fibers introduces new opportunities for surface engineering, while laser processing enables localized and controlled modification of the fiber surface without compromising the integrity of the bulk material. In this work, graphene-modified Kevlar^®^ KM2-600 fabrics were subjected to controlled laser processing under various parameter settings, and the resulting surface modifications were systematically analyzed. Three-dimensional laser microscopy was employed to characterize surface morphology and roughness, providing detailed insight into laser-induced topographical changes. The results demonstrate that optimized laser processing enables controlled surface restructuring while avoiding severe thermal damage, particularly when appropriate mechanical stabilization and focal conditions are maintained. Under identical laser processing conditions (Matrix II, q = 3.65 × 10^4^ W/cm^2^), the mean arithmetic roughness increased from 4.57 ± 1.04 µm for the unmodified fabric to 5.54 ± 1.05 µm for the graphene-modified fabric, while the mean root mean square roughness increased from 5.76 ± 1.41 µm to 6.95 ± 1.39 µm. These findings contribute to an improved understanding of laser–graphene–aramid interactions and provide a foundation for future studies addressing the potential functional implications of surface modification in lightweight protective textiles.

## 1. Introduction

Ballistic textiles play a critical role in modern military and personal protection systems due to their ability to dissipate impact energy [1,2], resist puncture [3], and reduce injuries caused by fragments or high-velocity projectiles [4]. Among these materials, para-aramid fibers such as Kevlar^®^ are widely recognized for their high strength-to-weight ratio [5], thermal resistance, and durability, making them essential components of body armor and protective equipment [6]. In composite systems, Kevlar^®^ fibers are commonly used as reinforcement materials in polymer matrices, where their performance is strongly influenced by fiber–matrix interfacial adhesion [5,6]. However, the inherently smooth and chemically inert surface of para-aramid fibers can limit interfacial adhesion and effective stress transfer, which has motivated the development of various surface modification techniques [7,8,9]. The molecular architecture of Kevlar^®^ consists of linear chains of para-substituted aromatic rings, which promote rigid, rod-like molecular alignment and enable the formation of liquid-crystalline polymer phases [10]. This highly ordered structure contributes to the high stiffness, tensile strength, and thermal stability that characterize para-aramid fibers, making them well suited for ballistic protection and aerospace applications [11]. With increasing global security demands, improving the performance of such fabrics without adding additional weight has become a key objective in material development. Recent advances in laser-based processing [12] have enabled new approaches for modifying Kevlar^®^ para-aramid surfaces, offering opportunities to modify surface characteristics. In parallel, recent studies have explored a wide range of surface modification strategies for para-aramid fibers aimed at improving interfacial performance in composite systems [7,9,13]. These approaches include plasma treatment, chemical functionalization, and the application of nano-scale coatings such as carbon-based nanomaterials. Among these, graphene has attracted considerable attention due to its high surface area, excellent thermal conductivity, and ability to modify surface energy and interfacial interactions [9,14]. These characteristics make graphene particularly suitable for surface engineering applications in high-performance textile systems. The deposition of graphene onto Kevlar^®^ fabric surfaces [15] further expands these possibilities due to its exceptional mechanical strength and potential to modify surface interactions.

However, achieving controlled surface modification remains challenging due to the localized and transient temperature rise generated within the laser interaction zone. Although Kevlar^®^ para-aramid fibers exhibit good intrinsic thermal stability and undergo thermal degradation only at elevated temperatures, as reported in thermogravimetric studies [16], excessive local energy input during laser irradiation may lead to undesired surface damage. Therefore, careful control of laser power density and scanning parameters is essential to enable surface restructuring while avoiding thermal degradation. Laser processing offers a highly localized and temporally confined energy input, which allows modification of the fiber surface without uniformly heating the bulk material. As a result, the temperature within the laser interaction zone can be controlled through appropriate selection of power density, scanning speed, and focal conditions, enabling surface modification of para-aramid fibers while preserving their intrinsic structural stability.

One of the emerging techniques for depositing graphene onto Kevlar is liquid-phase exfoliation (LPE), which has been increasingly reported in the literature for producing graphene-coated Kevlar-based materials [15,17,18,19,20]. In many of these approaches, the textile surface is laser-treated prior to graphene deposition [12]. CO_2_ lasers are commonly employed due to their suitable wavelength and adjustable energy output [21], enabling precise control of power density during surface modification [22]. Accurate tuning of laser parameters is required to promote controlled surface activation and surface modification while avoiding excessive local heating or surface damage.

In addition to the growing interest in graphene-based reinforcement strategies [15], recent studies have emphasized the importance of understanding micro- and nanoscale surface interactions in para-aramid composites [13]. Surface morphology, fiber roughness, and interfacial bonding [23] have been identified as key parameters that influence stress distribution and energy absorption during ballistic impact [24,25,26]. Laser-induced surface modification [27] provides a controllable approach for altering these parameters without the need for chemical treatments [7,28], which may degrade the fabric or introduce unwanted residues. Furthermore, the development of advanced characterization techniques such as atomic force microscopy, three-dimensional profilometry, and high-resolution optical microscopy has enabled detailed evaluation of surface topography changes following laser irradiation [12,29,30,31]. Despite these advances, comprehensive studies that combine graphene modification with post-deposition laser processing remain limited [12]. This highlights the need for studies that systematically investigate how graphene present on the fiber surface influences subsequent laser–material interaction under controlled conditions.

Current studies primarily examine Kevlar-based fabrics that are first laser-processed and subsequently coated with graphene [12,15,32], or Kevlar^®^ fabrics that are coated with graphene without any laser-assisted surface preparation [33]. In contrast, the behavior of para-aramid fabrics that already contain graphene deposited via the liquid-phase exfoliation (LPE) method and are then exposed to laser processing has not yet been systematically examined. The lack of research on this processing sequence creates a notable gap in understanding how graphene influences the laser–aramid interaction mechanism [15,33], surface topography, and resulting mechanical properties. Addressing this gap is essential for optimizing next-generation ballistic materials and establishing reliable processing protocols.

In this work, graphene-modified KM2-600 Kevlar^®^ fabrics were subjected to controlled laser processing to investigate the influence of pre-applied graphene on laser-induced surface morphology and topographical evolution. The purpose of this study is to investigate how a pre-deposited graphene surface layer influences subsequent laser–material interaction under controlled processing conditions. In this context, graphene is considered as a surface-modifying layer that can influence local energy absorption and surface restructuring during laser irradiation, as reported in previous studies [12], while laser processing is used as a controlled physical method for generating micro-scale surface topography changes. The proposed processing sequence was selected to investigate the combined effect of graphene modification and laser irradiation on surface morphology. Unlike conventional approaches in which graphene is deposited after laser-based surface treatment [12], the present study applies laser irradiation directly to graphene-modified para-aramid fibers, thereby enabling systematic investigation of the graphene-mediated laser–material interaction mechanism [14,27,34]. The processed samples were analyzed using three-dimensional laser microscopy to evaluate topographical changes and their potential relevance for surface-related interactions and material behavior.

## 2. Materials and Methods

### 2.1. Material Characteristics of Kevlar^®^

The present study focuses on the experimental evaluation of graphene-modified para-aramid fabric (Kevlar^®^ KM2-600), which is commonly used in ballistic applications. Commercial Kevlar^®^ KM2 fabric woven from ballistic-grade para-aramid fibers with a linear density of 600 dtex was selected as the base material for this study. This material was selected due to its widespread use in ballistic protection applications, where high strength-to-weight ratio, energy absorption capability, and structural stability are critical performance requirements [4,5,6]. The fabric features a plain-weave architecture with a balanced thread density of 11.2 cm^−1^ in both warp and weft directions, providing uniform yarn distribution and a mechanically stable textile structure. Such fabric architecture is advantageous for controlled laser surface processing and for ensuring reproducible surface modification.

According to the manufacturer’s specifications, the nominal fabric thickness is 0.23 ± 0.02 mm, while the areal density is 146 g/m^2^. The supplied material included a standard ultraviolet (UV) protection finishing representative of commercially deployed ballistic textiles. Prior to graphene coating, the fabrics were conditioned and prepared under controlled conditions, as described in the following sections.

The key physical and mechanical properties of Kevlar^®^ KM2-600 fibers are summarized in Table 1. These properties, including high tensile strength and elevated Young’s modulus, are representative of advanced para-aramid materials used in ballistic systems.

### 2.2. Sample Preparation and Graphene Coating Procedure

Before applying the graphene coating, the Kevlar^®^ fabric was subjected to a multi-stage cleaning procedure to remove finishing agents and surface contaminants. This cleaning procedure follows commonly reported surface preparation methods for aramid fabrics prior to coating and was adapted to ensure consistent and reproducible surface conditions for graphene deposition [18]. The samples were immersed in acetone for 2 h, rinsed thoroughly, and air-dried to ensure a clean and uniform surface suitable for subsequent modification [18]. All fabric specimens were cut to a size of 250 × 250 mm and conditioned under standard laboratory conditions prior to processing. This preparation step was essential to minimize variability between specimens and to ensure consistent surface condition across all tested samples.

The graphene coating was deposited on the cleaned Kevlar^®^ fabric using a dip-coating technique, in which the fabric samples were immersed in a graphene dispersion and subsequently withdrawn at a controlled rate to allow uniform coating formation, followed by thermal consolidation to achieve an even distribution of graphene across the fiber network [18,19]. The dip-coating approach was selected due to its simplicity, scalability, and ability to achieve uniform coating on complex textile structures. This method allows the graphene nanoplatelets to form a continuous, uniform layer along the fiber surfaces, promoting uniform coating distribution across the textile structure. After coating, the samples were subjected to thermal treatment at 60 °C for 1 h to stabilize the graphene layer on the para-aramid fiber surface [9]. The subsequent thermal treatment step also improves coating consistency and ensures repeatable processing quality for subsequent laser treatment and characterization.

### 2.3. Laser Setup

Laser processing was carried out using a CO_2_ Suntop ST-CC9060 system equipped with a continuous-wave source operating at a wavelength of 10,640 nm. CO_2_ laser systems of this type are widely applied for surface modification and texturing of polymer-based and textile materials due to their stable output and controllable processing parameters. The unit provides a maximum output power of 100 W and includes a working platform measuring 900 × 600 mm, allowing the treatment of relatively large textile samples. The positioning accuracy of the system is 0.02 mm, enabling precise control over the irradiation path during processing.

The scanning speed can be varied between 0 and 1000 mm/s, allowing adjustment of the energy input according to the specific processing requirements. To ensure stable operation, the laser system is supported by an integrated water-cooling unit that maintains the temperature of the optical components within the recommended limits. The device is classified as a Class 4 laser; therefore, all procedures were conducted in accordance with appropriate laser safety protocols. The system accepts multiple data formats, including DXF and BMP, facilitating flexible preparation of scanning patterns. The total electrical power consumption of the setup is 1500 W. The main technical specifications of the CO_2_ laser system are summarized in Table 2.

**Figure 1:**
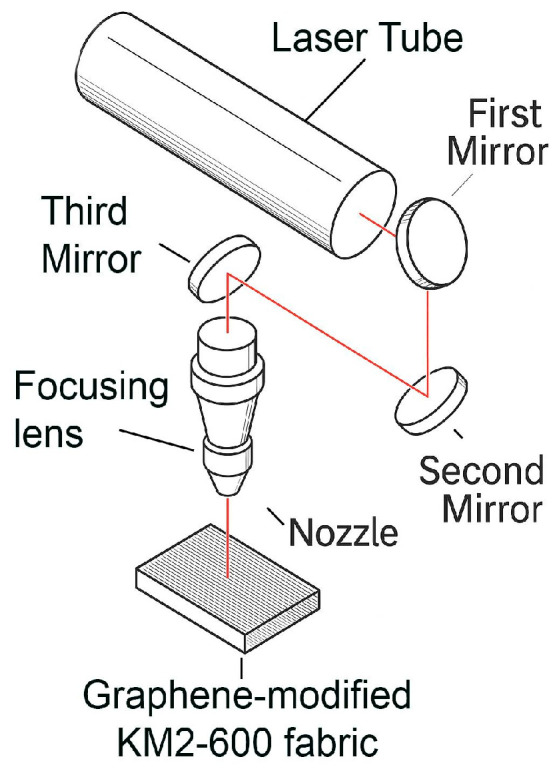
CO_2_ laser experimental setup schematic for sample processing.

During laser processing, each graphene-modified KM2-600 specimen was placed flat on the working platform and secured along the edges to prevent movement during scanning. This fixation method helped maintain a constant focal distance and preserved the natural in-plane tension of the fabric. The controlled positioning minimized vibration and ensured uniform energy delivery across the irradiated area. A schematic of the CO_2_ laser system used in this study is presented in Figure 1.

This laser configuration was consistently applied to all specimens throughout the study to ensure comparable processing conditions. For the investigated processing conditions, the applied laser powers were approximately 8.1 W (Matrix I), 8.3 W (Matrix II), and 8.5 W (Matrix III), corresponding to power density values of 3.57 × 10^4^, 3.65 × 10^4^, and 3.74 × 10^4^ W/cm^2^, respectively. The setup provided stable and repeatable irradiation parameters for subsequent surface modification experiments. In addition to the absolute laser power values, the laser power density was considered to provide a more general description of the applied irradiation conditions. The effective laser spot diameter used for the power density calculation was 0.017 cm (0.17 mm). The power density q was calculated as the ratio between the laser output power P and the effective irradiated area S on the fabric surface, according to Equation (1):

(1) $$q=\frac{P}{S},$$

where P is the applied laser power (W) and S represents the laser beam cross-sectional area at the focal plane. Assuming an approximately circular laser spot at the focal plane, the irradiated area was calculated according to Equation (2):

(2) $$S=\pi (\frac{d}{2}{)}^{2},$$

where d denotes the effective laser spot diameter. The calculated power density values were subsequently used for the interpretation of surface roughness and colorimetric changes presented in Section 3.

### 2.4. Laser Microscopy Analysis

The surface morphology and topographical features of the graphene-modified KM2-600 para-aramid fabric were examined using a 3D laser scanning microscope (Olympus LEXT OLS5000, Olympus Corporation, Tokyo, Japan). This technique enables non-contact, high-resolution characterization of textile surfaces and is particularly suitable for evaluating laser-induced surface modifications for various material systems [37]. Measurements were performed under high-magnification optical conditions to capture detailed surface features at the micro-scale. The microscope was operated using an objective with a numerical aperture of 0.6 and a working distance of approximately 1 mm, corresponding to a lateral resolution of approximately 0.4 μm. The effective focal depth was approximately 1.8 μm, with a laser spot diameter of ~0.8 μm, enabling precise acquisition of three-dimensional surface profiles. The scanned surface area for each measurement was 640 × 640 μm^2^.

The acquired 3D datasets were used to evaluate surface topography, laser-induced structural changes, and local surface irregularities of the untreated and laser-processed fabric regions. All measurements were performed under identical optical and acquisition conditions to ensure consistency and comparability between different samples.

Quantitative surface roughness parameters were obtained from the acquired three-dimensional surface datasets. The arithmetic mean height (Sa) and the root mean square height (Sq) were determined using the microscope software and evaluated in accordance with established areal surface texture characterization methodologies [38,39,40,41,42] and ISO 25178 [43]. For each sample condition, multiple measurement areas were analyzed, and the same evaluation procedure was applied to ensure consistency and comparability between untreated and laser-processed fabric regions. For each sample condition, five independent surface areas were analyzed (*n* = 5).

### 2.5. Colorimetric Analysis (CIE L*a*b*)

Colorimetric changes induced by laser processing were evaluated using the CIE L*a*b* color space [44,45], which is widely applied for quantitative assessment of surface color variations [46]. This color model represents color information in a three-dimensional space defined by lightness (L*), red–green chromaticity (a*), and blue–yellow chromaticity (b*), allowing objective comparison of color changes before and after surface modification.

For each sample condition, colorimetric values were obtained from representative surface regions of untreated and laser-processed fabric areas under identical acquisition conditions. The differences in individual color channels (ΔL*, Δa*, Δb*) were calculated to characterize changes in surface lightness and chromaticity resulting from laser irradiation.

The overall color difference between untreated and laser-processed regions was quantified using the total color difference parameter ΔE*, calculated according to Equation (3):

(3) $$\Delta E^{*}=\sqrt{\left[{\left(\Delta L^{*}\right)}^{2}\;+\;{\left(\Delta a^{*}\right)}^{2}\;+\;{\left(\Delta b^{*}\right)}^{2}\right]},$$

This parameter provides a single quantitative metric describing the magnitude of color change induced by laser processing. The applied approach enables consistent comparison of colorimetric variations between different processing conditions and complements the surface topography analysis obtained by 3D laser microscopy.

## 3. Results

### 3.1. Surface Morphology After Laser Processing

The initial assessment of laser-induced surface modifications was performed through visual inspection of the fabric surfaces.

Representative surface images were acquired to illustrate the overall appearance of the para-aramid fabrics before and after laser processing under different material and processing conditions. This qualitative comparison provides an overall visual overview of laser-induced changes prior to detailed micro-scale analysis. In this study, Matrix I, Matrix II, and Matrix III correspond to predefined laser processing parameter sets with increasing laser power density, while all other processing parameters were kept constant.

**Figure 2:**
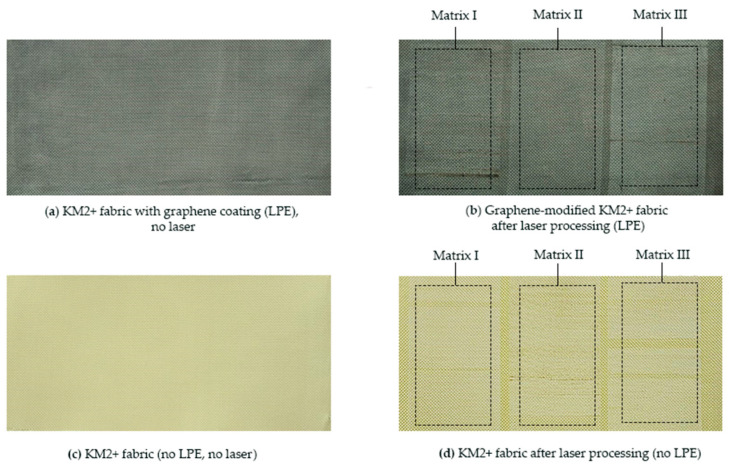
Visual appearance of para-aramid fabric surfaces prior to and following laser processing. (**a**) KM2+ fabric with graphene coating (LPE) before laser processing; (**b**) graphene-modified KM2+ fabric after laser processing; (**c**) KM2+ fabric without LPE before laser processing; and (**d**) KM2+ fabric after laser processing without LPE. Laser-marked surface regions corresponding to Matrix I–III represent increasing laser power density under identical processing conditions.

As illustrated in Figure 2, laser processing results in distinct visual differences between untreated and laser-treated fabric surfaces. The presence of multiple laser-marked surface regions on the same specimen enables direct comparison of surface appearance under increasing laser power density. To further examine the observed surface features and assess structural changes at smaller length scales, selected regions from each laser-marked surface region were subsequently analyzed using three-dimensional laser microscopy.

**Figure 3:**
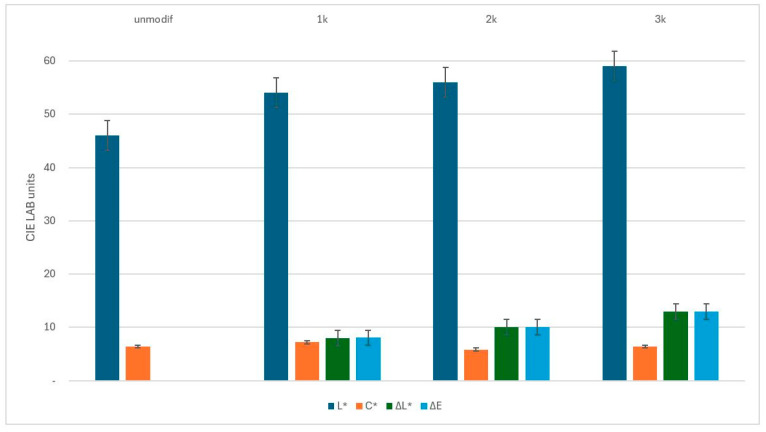
Variation in CIELAB color parameters (L*, a*, b*, C*) and total color difference (ΔE*) of graphene-modified KM2-600 fabric as a function of laser power density. Matrix I (q_1_ ≈ 3.57 × 10^4^ W/cm^2^), Matrix II (q_2_ ≈ 3.65 × 10^4^ W/cm^2^), and Matrix III (q_3_ ≈ 3.74 × 10^4^ W/cm^2^) correspond to increasing laser power density.

As shown in Figure 3, laser processing leads to clear and systematic changes in the surface color of the graphene-modified KM2-600 fabric. A clear trend is observed across Matrix I–III, where increasing laser power density results in progressively greater changes in surface color. The results demonstrate that surface color parameters vary consistently with increasing laser power density. In Figure 3, the surface regions are labeled as 1k, 2k, and 3k, corresponding to Matrix I, Matrix II, and Matrix III, respectively. These labels represent increasing laser power density, while all other processing conditions were kept constant.

With increasing laser power density from q_1_ ≈ 3.57 × 10^4^ W/cm^2^ (Matrix I) to q_3_ ≈ 3.74 × 10^4^ W/cm^2^ (Matrix III), a gradual increase in the lightness parameter L* is observed. This indicates that the initially dark graphene-coated fabric surface becomes progressively lighter as the applied laser power density increases as shown in Figure 2b. These changes are associated with laser-induced surface modification, including changes in surface roughness, local morphology, and possible redistribution of the graphene coating. The observed increase in lightness is in good agreement with the visual appearance of the laser-marked areas, where higher power densities result in brighter surface regions. The chroma parameter (C*) remains within a relatively narrow range across all laser-processed surface regions, suggesting that laser irradiation primarily affects lightness (L*) rather than causing pronounced changes in chroma (C*). In contrast, the total color difference ΔE* increases steadily with increasing laser power density, confirming that laser processing produces measurable and distinguishable color changes relative to the unmodified reference surface.

Overall, the colorimetric analysis shows that laser power density is a key parameter governing the colorimetric response of graphene-modified para-aramid fabrics.

This relationship highlights the sensitivity of the graphene-coated surface to localized effects of laser irradiation, which influence both surface morphology and surface characteristics. Even small increases in power density led to observable and quantifiable changes in surface color, indicating controlled laser–material interaction under the applied processing conditions.

### 3.2. Three-Dimensional Laser Microscopy Analysis of Surface Morphology

Micro-scale surface analysis in this study focused on laser-processed surface regions of the para-aramid fabric, as these regions represent the locations where controlled laser–material interaction produces laser-induced surface modifications. The selected analysis regions were aligned with the scanning direction to ensure consistent comparison, although laser-induced modifications occur across fibers in both warp and weft directions within the irradiated area. In several previous studies, laser surface modification has been applied as an initial step, followed by the deposition or formation of a graphene-based coating on the laser-treated surface [12]. In the present work, a different processing sequence is employed, in which graphene is first deposited onto the Kevlar^®^ fabric surface, forming a modified surface layer prior to laser exposure. Subsequent laser processing is applied directly to the graphene-modified fabric. This enables interaction between the laser irradiation, the graphene coating, and the underlying para-aramid fiber structure under controlled processing parameters. The untreated Kevlar fabric without graphene modification was used to define the initial reference surface state. This reference state allows direct comparison of surface morphology before and after laser processing under identical experimental conditions. Comparative evaluation was performed between laser-marked regions on graphene-modified and unmodified fabrics under identical laser processing conditions. This approach allows systematic investigation of laser-induced surface features as a function of laser power density while isolating the influence of graphene modification.

**Figure 4:**
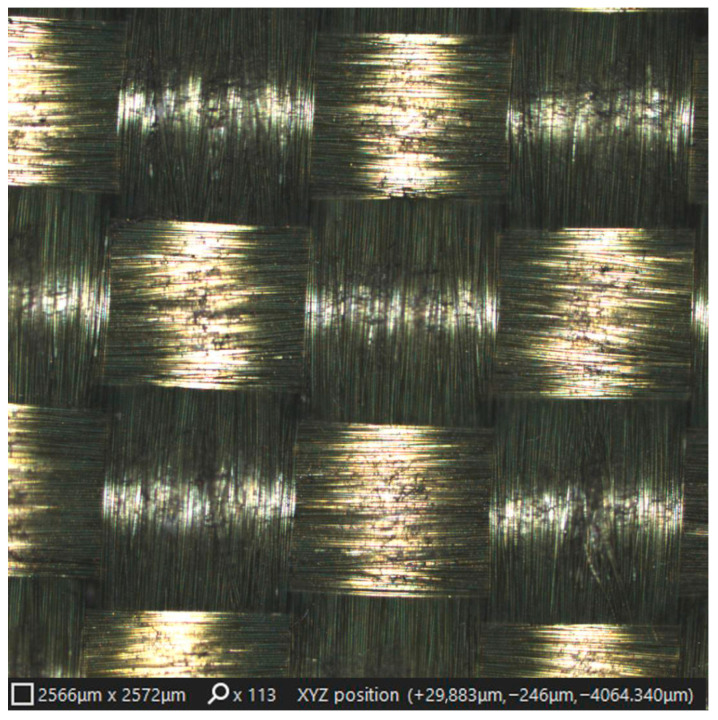
Yarn and weave structure of graphene-modified KM2-600 para-aramid Kevlar^®^ fabric.

The characteristic yarn and weave structure of the KM2 para-aramid fabric is illustrated in Figure 4. The image highlights the filament bundle arrangement and the inherent surface topography of the woven fabric, providing structural context for the subsequent micro-scale analysis of laser-processed surface regions. This initial structural overview allows the intrinsic textile morphology to be distinguished from surface features arising due to laser irradiation and graphene modification in later stages of the analysis.

To investigate laser-induced surface modifications in greater detail, representative laser-processed surface regions were further examined using three-dimensional laser microscopy. Confocal laser scanning images and corresponding three-dimensional surface maps were acquired from identical surface locations to enable combined qualitative and quantitative assessment of laser–material interactions. The use of complementary imaging modes provides detailed insight into fiber morphology, surface relief, and local topographical variations. This combined approach forms the basis for systematic comparison of laser-processed regions under different material conditions and laser power densities presented in the following sections. The analyzed confocal microscopy images represent localized regions selected from larger laser-processed surface areas.

**Figure 5:**
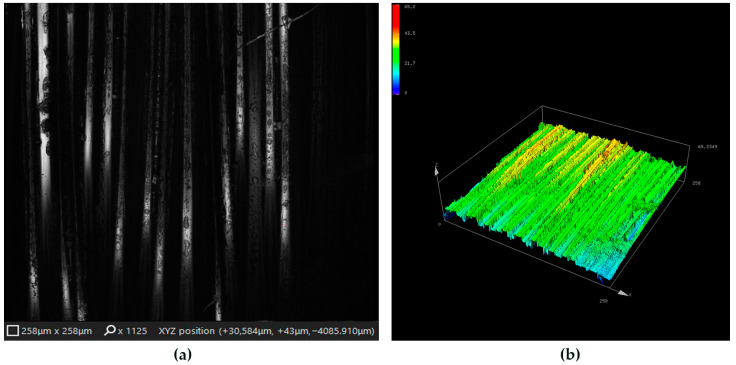
Representative morphology of a laser-processed surface region corresponding to Matrix II on KM2-600 fabric: (**a**) confocal image and (**b**) corresponding 3D surface height map acquired from the same region. Processing parameters: (q = 3.65 × 10^4^ W/cm^2^, Δx = 80 µm).

As shown in Figure 5, laser irradiation of the unmodified KM2-600 fabric induces clearly distinguishable surface features aligned with the fiber direction. The confocal laser scanning image (Figure 5a) reveals localized modifications along individual filament bundles, indicating an increase in surface roughness without visible fiber breakage or melting. These changes suggest controlled laser–material interaction confined primarily to the fiber surface. 

The corresponding three-dimensional surface map (Figure 5b) shows height variations that follow the woven fiber architecture of the KM2-600 fabric. Areas with increased surface relief are visible as regions with higher color intensity [40,47], indicating localized increases in surface roughness. These features are associated with laser-induced surface restructuring of the para-aramid fibers caused by localized laser–material interaction. Importantly, no evidence of severe fiber damage or material removal is observed. The resulting increase in surface roughness represents a controlled surface modification, while maintaining the structural integrity of the fabric. To further investigate the effect of graphene modification on laser-induced surface morphology, additional micro-scale surface analysis was performed on graphene-modified KM2-600 fabric subjected to laser processing under comparable irradiation conditions. In this case, the graphene layer is already present on the para-aramid surface prior to laser exposure, which alters the initial surface state before laser irradiation.

Representative confocal laser scanning images and corresponding three-dimensional surface height maps were acquired from laser-marked surface regions on graphene-modified fabric using the same optical settings and scanning parameters as for the unmodified reference. This approach allows direct visual and topographical comparison between laser-processed surfaces with and without graphene modification.

**Figure 6:**
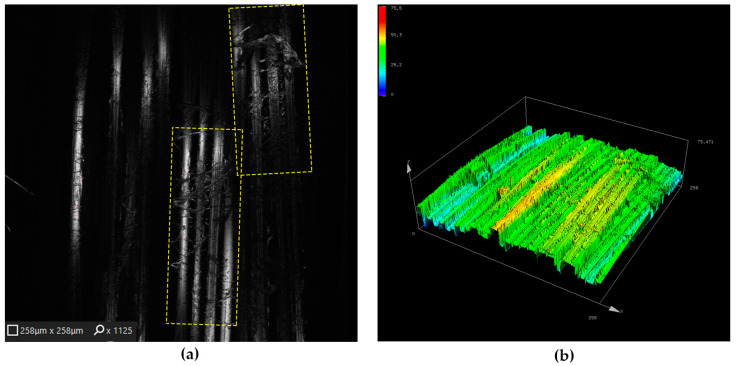
Representative surface morphology of a laser-processed surface region corresponding to Matrix II on graphene-modified KM2-600 para-aramid fabric: (**a**) confocal laser scanning image and (**b**) corresponding three-dimensional surface height map acquired from the same region. The yellow dashed boxes in Figure 6a indicate representative regions exhibiting localized surface restructuring and graphene-associated morphological features after laser processing. Laser processing parameters: power density q = 3.65 × 10^4^ W/cm^2^ and raster step Δx = 80 µm.

A representative laser-processed surface region on graphene-modified KM2-600 fabric is shown in Figure 6, providing the basis for comparative assessment of surface morphology and roughness evolution relative to the unmodified fabric presented in Figure 5.

Comparison of the micro-scale surface images presented in Figure 5 and Figure 6 reveals distinct differences in the para-aramid yarn morphology resulting from graphene modification prior to laser processing. Compared to the unmodified fabric, the graphene-modified sample exhibits more pronounced surface restructuring under identical laser conditions. At the microscopic level, the graphene-modified KM2-600 fabric exhibits a noticeably altered surface structure compared to the unmodified reference following laser irradiation. As observed in the confocal laser scanning image in Figure 6a, graphene deposited via the liquid-phase exfoliation (LPE) method [15] forms a surface coating, with associated morphological features observed along individual filaments and, in certain regions, appearing to extend between neighboring filaments within the yarn structure after laser exposure. This suggests that laser processing may promote closer interaction between the graphene-modified surface layer and para-aramid fibers, leading to localized surface features that involve multiple filaments rather than isolated fiber surfaces. 

Such graphene-assisted inter-fiber connectivity has been reported in the literature to enhance load transfer in Kevlar^®^ and para-aramid-based textile systems [12,48]. This effect has been associated with increased resistance to fiber pull-out under mechanical loading. In the present study, similar micro-scale surface features are observed. While the observed surface features are consistent with mechanisms reported to enhance inter-fiber load transfer, direct mechanical testing was beyond the scope of the present study and will be addressed in future work.

Further comparison of the corresponding three-dimensional surface height maps (Figure 6b versus Figure 5b) shows that laser-processed regions on graphene-modified fabric exhibit more pronounced surface relief than the unmodified reference under identical processing conditions. Notably, both samples were processed under identical laser irradiation conditions, including a power density of q = 3.65 × 10^4^ W/cm^2^, a raster step of Δx = 80 µm, and a scanning speed of 150 mm/s, ensuring that the observed differences are attributable to the graphene modification rather than variations in laser parameters. The graphene-modified surface displays a higher density of surface peaks and more pronounced valleys, indicating an increased development of surface roughness.

This enhanced surface roughness reflects the combined influence of laser-induced surface restructuring and the presence of the graphene coating, which alters the local topography of the para-aramid yarn surface. In comparison with the unmodified fabric, the graphene-modified sample exhibits a more complex and heterogeneous surface morphology. 

These observations suggest that the presence of graphene may promote a more effective laser–surface interaction under identical irradiation conditions. This effect may be associated with enhanced local energy absorption and redistribution at the graphene–fiber interface, leading to more pronounced surface restructuring. To quantitatively support these qualitative observations, the evolution of surface roughness was further evaluated using areal roughness parameters, as presented in the following section.

### 3.3. Quantitative Surface Roughness Analysis

To quantitatively support the qualitative observations obtained from confocal and three-dimensional surface images, areal surface roughness parameters were evaluated for laser-processed KM2-600 para-aramid fabrics. The analysis was focused on laser-marked surface regions, where controlled laser–material interaction induced measurable changes in surface topography. Three-dimensional surface height maps acquired by confocal laser scanning microscopy were used to extract standardized areal roughness parameters in accordance with ISO 25178.

The values reported in Table 3 are presented as mean ± SD (*n* = 5) to account for local textile heterogeneity. The laser-processed KM2-600 fabric without graphene modification (Matrix II, q = 3.65 × 10^4^ W/cm^2^) was used as the reference to establish baseline roughness values, while comparative analysis was performed for graphene-modified samples processed under identical laser conditions. The arithmetic mean height (Sa) and root mean square height (Sq) were selected as representative areal roughness parameters to describe changes in surface amplitude and overall height distribution resulting from laser processing.

Based on the average data presented in Table 3, laser processing results in an overall increase in surface roughness for graphene-modified KM2-600 fabrics compared to the unmodified reference under identical irradiation conditions. This direct comparison suggests that graphene modification enhances surface roughness development under identical laser processing conditions. The mean Sa value increases from 4.57 µm for the unmodified fabric to 5.54 µm for the graphene-modified sample, while Sq increases from 5.76 µm to 6.95 µm. Despite local variability inherent to woven textile structures, the consistent upward trend across multiple measurement areas indicates that graphene modification contributes to enhanced development of surface height variations during laser processing. 

**Figure 7:**
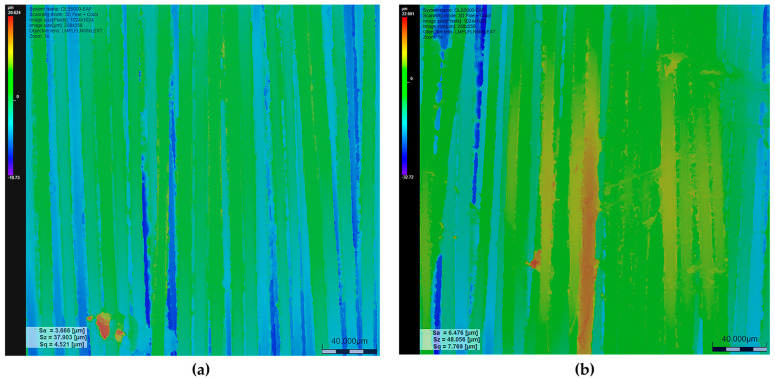
Representative surface roughness maps of laser-processed KM2-600 para-aramid fabric (Matrix II): (**a**) unmodified fabric and (**b**) graphene-modified fabric, extracted from confocal laser scanning data. Laser processing parameters: power density q = 3.65 × 10^4^ W/cm^2^, raster step Δx = 80 µm, scanning speed 150 mm/s.

To further illustrate these micro-scale features, representative surface roughness maps are presented in Figure 7. The untreated fabric (Figure 7a) corresponds to a representative measurement area with Sa = 3.666 µm and Sq = 4.521 µm, while the graphene-modified sample (Figure 7b) shows a representative area with Sa = 6.476 µm and Sq = 7.769 µm under identical processing conditions.

As illustrated in Figure 7, the surface roughness maps clearly demonstrate differences in surface height distribution between the unmodified and graphene-modified KM2-600 fabrics processed under identical laser irradiation conditions. In Figure 7a, corresponding to the unmodified fabric, the surface is predominantly characterized by blue and green color regions, indicating relatively lower height variations and a more uniform surface profile. The surface relief follows the yarn direction with limited development of pronounced surface peaks or deep valleys.

In contrast, the graphene-modified fabric shown in Figure 7b exhibits a wider distribution of yellow and red regions, corresponding to areas of increased surface height and deeper surface depressions. This indicates a higher density of surface peaks and valleys and a more heterogeneous surface topography relative to the unmodified reference, which is consistent with the quantitative roughness data presented in Table 3, where the graphene-modified fabric exhibits higher mean Sa and Sq values than the untreated sample. While local textile heterogeneity contributes to variability across individual measurement areas, the overall trend confirms an increase in surface roughness following graphene modification and subsequent laser processing. This increase is relevant because changes in surface roughness may influence interfacial interaction through mechanical interlocking, which may affect load transfer in textile-based systems. 

In textile-based composite systems, increased surface roughness may promote mechanical interlocking between fibers and coatings, which may influence load transfer under mechanical loading. From a practical perspective, such surface modifications may be relevant for ballistic protection systems and high-performance composite materials, where enhanced interfacial interaction may contribute to improved energy absorption and structural stability. Therefore, the analysis was focused on identifying consistent trends under controlled conditions rather than on formal statistical hypothesis testing.

The roughness maps further demonstrate that graphene modification enhances the development of laser-induced surface relief under identical processing parameters. The combined effect of laser irradiation and the presence of the graphene coating promotes more pronounced surface structuring, resulting in a rougher and more complex topography than observed for the unmodified KM2-600 fabric. These observations provide consistent qualitative and quantitative evidence that graphene modification intensifies laser-induced surface morphology evolution. 

Overall, the combined colorimetric and three-dimensional surface roughness analyses demonstrate that laser processing induces systematic and controllable modifications of the KM2-600 fabric surface, with graphene modification significantly influencing the resulting surface morphology. The implications of these surface changes and their relevance to material performance are discussed in the following section.

## 4. Discussion

Laser-based surface modification of para-aramid fabrics is characterized by a relatively narrow processing window, within which beneficial surface restructuring can be achieved without inducing structural damage. The results presented in Section 3.1, Section 3.2 and Section 3.3 demonstrate that, within the selected range of laser power density and scanning parameters, controlled laser irradiation enables systematic modification of surface morphology and roughness. These effects are particularly pronounced when laser processing is combined with prior graphene surface modification, which alters the initial surface state and influences the subsequent laser–material interaction.

**Figure 8:**
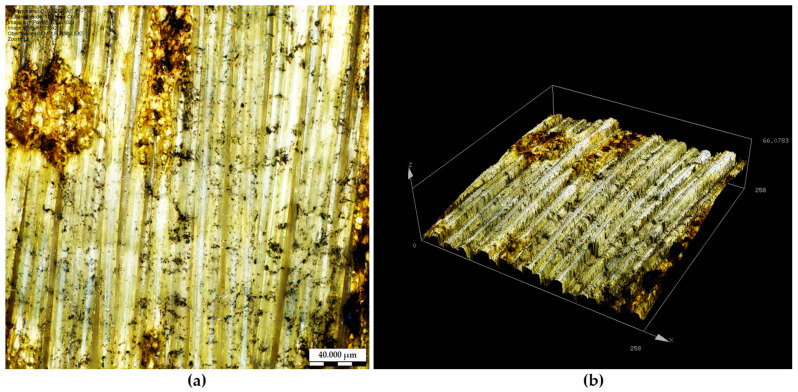
Locally damaged surface region observed during laser processing of KM2-600 para-aramid fabric at elevated laser power density (q_3_ ≈ 3.74 × 10^4^ W/cm^2^): (**a**) optical micrograph and (**b**) corresponding three-dimensional surface height map acquired from the same region.

Figure 8 presents optical and three-dimensional images of a locally damaged surface region observed during laser processing of KM2-600 para-aramid fabric at an elevated laser power density (q_3_ ≈ 3.74 × 10^4^ W/cm^2^). Localized surface defects were occasionally identified during the experimental campaign, particularly at higher power density levels and under non-ideal sample fixation conditions. These defects are primarily characterized by surface waviness, local deformation, and irregular height variations rather than complete fiber ablation or melting. The corresponding three-dimensional surface height map further highlights the amplitude of these local height variations, confirming that the observed damage is confined to surface restructuring rather than bulk material removal.

Such surface defects can be attributed, among other contributing factors, to local defocusing (Δf) of the laser beam caused by insufficient mechanical stabilization and the inherently uneven surface of the woven textile. In flexible para-aramid fabrics, local out-of-plane displacement may occur during laser scanning, resulting in deviations of the material surface relative to the nominal focal plane. As described in the literature, laser processing under defocused conditions modifies the effective interaction between the laser beam and the material surface, thereby altering the local energy distribution and increasing the likelihood of localized overheating or surface deformation, even when nominal laser parameters remain constant [49,50,51].

It is important to emphasize that the damaged regions shown in Figure 8 do not represent the dominant surface response under optimized processing conditions. Instead, they illustrate the sensitivity of laser treatment applied to flexible fibrous substrates to mechanical stabilization and focal positioning. When appropriate fixation and controlled processing conditions are maintained, the laser-induced surface modifications observed in this study remain confined primarily to surface restructuring rather than material degradation. In contrast to the isolated defects observed under non-ideal conditions, the majority of laser-processed regions exhibit controlled roughness enhancement and modified surface topography without significant fiber damage. This behavior is particularly evident for graphene-modified KM2-600 fabrics, where the combined presence of graphene coating and laser irradiation promotes more pronounced surface relief while preserving fiber integrity. This behavior is associated with the interaction between laser irradiation and the graphene-modified surface under the applied processing conditions. 

The direct comparison between unmodified and graphene-modified fabrics processed under identical laser conditions suggests that the presence of graphene influences the laser–material interaction and contributes to enhanced surface structuring. Overall, the discussion confirms that laser power density, surface modification sequence, and mechanical stabilization of the textile are critical parameters governing the quality and reproducibility of laser-induced surface modifications. While the present work focuses on morphological and topographical characterization, the observed surface features indicate consistent changes in surface morphology and roughness under the applied processing conditions. Future studies will therefore address the mechanical implications of these surface modifications, including yarn pull-out resistance and interfacial performance, to further elucidate the role of graphene-assisted laser processing in para-aramid textile systems.

## 5. Conclusions

In this study, the influence of pre-applied graphene on laser-induced surface morphology and topographical evolution of KM2-600 para-aramid fabrics was systematically investigated. The results demonstrate that controlled laser processing enables precise modification of the fabric surface without inducing significant structural damage to the fibers.

Comparative analysis revealed that graphene-modified fabrics exhibit increased surface roughness under identical processing conditions. The arithmetic mean roughness (Sa) increased from 4.57 ± 1.04 µm to 5.54 ± 1.05 µm, while the root mean square roughness (Sq) increased from 5.76 ± 1.41 µm to 6.95 ± 1.39 µm. 

The results indicate that the presence of graphene affects the laser–material interaction, leading to more pronounced surface structuring under identical processing conditions. These findings contribute to a better understanding of laser–graphene–aramid interactions and provide a basis for further investigation of surface modification in para-aramid materials. However, the evaluation of mechanical and ballistic performance was beyond the scope of the present study and will be addressed in future work. Future work will focus on mechanical characterization and advanced material analysis to evaluate the functional performance of the modified fabrics.

## Figures and Tables

**Table 1:** Mechanical and physical properties of Kevlar^®^ KM2-600 fibers and fabric.

Parameter	Value	Reference
Fiber type	Kevlar^®^ KM2 (ballistic-grade para-aramid)	SAATI
Linear density	600 dtex	Manufacturer
Weave structure	Plain weave, style 310	Manufacturer
Thread density	11.2 cm^−1^ (warp/weft)	Manufacturer
Fabric areal density	146 g/m^2^	Manufacturer
Fabric thickness	0.23 ± 0.02 mm	Manufacturer
Fiber diameter	~12 μm	[35,36]
Young’s modulus	84.62 ± 4.18 GPa	[35,36]
Tensile strength	3.88 ± 0.40 GPa	[35,36]
Failure strain	4.52% ± 0.37%	[35,36]
Specimen size	250 × 250 mm	Measured

**Table 2:** Technical specifications of the Suntop ST-CC9060 CO_2_ laser system.

Parameter	Specification
Laser wavelength	10,640 nm
Maximum output power	100 W
Operating mode	Continuous wave (CW)
Working area	900 × 600 mm
Positioning accuracy	0.02 mm
Adjustable scanning speed	0–1000 mm/s
Safety classification	Class 4
Cooling method	Water-cooled system
Supported input formats	DXF, BMP
Total electrical power consumption	1500 W

**Table 3:** Areal surface roughness parameters (Sa and Sq) of KM2-600 para-aramid fabric after laser processing, with and without graphene modification (mean ± SD, *n* = 5), evaluated by three-dimensional laser microscopy.

Sample	Graphene	Matrix	q (W/cm^2^)	Sa (µm)	Sq (µm)
KM2-600	-	II	3.65 × 10^4^	4.57 ± 1.04	5.76 ± 1.41
KM2-600	Modified	II	3.65 × 10^4^	5.54 ± 1.05	6.95 ± 1.39

## Data Availability

The original contributions presented in this study are included in the article. Further inquiries can be directed to the corresponding author.

## DURC Statement

Current research is limited to materials engineering and laser surface modification of para-aramid textiles for protective applications, which is beneficial for improving material performance and surface functionality and does not pose a threat to public health or national security. The authors acknowledge the potential dual-use nature of advanced material processing technologies and confirm that all necessary precautions have been taken to prevent potential misuse. The experiments were conducted in accordance with relevant institutional and ethical research standards. The authors support responsible research practices, transparency, and regulatory compliance to minimize misuse risks and promote beneficial scientific outcomes.
